# Knowledge and Attitudes of Dentists with Respect to the Risks of Blood-Borne Pathogens—A Cross-Sectional Study in Poland

**DOI:** 10.3390/ijerph14010069

**Published:** 2017-01-12

**Authors:** Anna Garus-Pakowska, Mariusz Górajski, Franciszek Szatko

**Affiliations:** 1 Department of Hygiene and Health Promotion, Medical University of Lodz, Lodz 90-647, Poland; franciszek.szatko@umed.lodz.pl; 2 Department of Econometrics, University of Lodz, Lodz 90-214, Poland; mariuszg@math.uni.lodz.pl

**Keywords:** dentists, occupational exposure, blood and potentially infectious material, knowledge, attitudes, prevention

## Abstract

*Background*: To analyze dentists’ knowledge of blood-borne infections, their attitudes towards infected patients, and to determine the frequency of the contact with infectious material; *Methods*: We surveyed 192 dentists using an anonymous questionnaire. *Results*: Only a quarter of dentists responded correctly to all questions. 96% of the examined dentists confirmed that they were more cautious during treatment of patients with HBV, HCV and HIV. 25% of all respondents refuse to help infected patients due to concerns about their own health. The dentists occasionally removed protective clothing to make it “easier” to perform specific procedures. The dentists experienced contact with infectious material most frequently by splashes onto the conjunctiva or as a result of superficial injuries. The risk of injury by a medical tool increased with the years of employment. Re-capping needles was associated with an increased risk of injury; *Conclusions*: Despite the widespread tolerance of people infected with blood-borne viruses and the well-proven low infection risk to medical personnel, dentists continue to be prejudiced and concerned about their own health and may refuse to treat infected patients. It may be assumed that the proportion of refusing treatment is even greater. This attitude should imply the implementation of training in the field of pathogen transmission and the real risk of infection.

## 1. Introduction

In the workplace, health care workers are exposed to a number of physical, chemical, biological and psychosocial risk factors [1,2]. Due to the nature of the work (standing or forced body posture), most frequent health problems reported by the dentists include musculoskeletal diseases [3,4,5]. In Poland in 2012–2014, chronic diseases of the musculoskeletal system were—apart from chronic diseases of the nervous system—the most common occupational diseases reported by dentists [6,7,8]. However, dentists must also be aware of the risk of infections by various microorganisms, such as HBV, HCV, HIV, *Mycobacterium tuberculosis*, herpes simplex virus, mumps virus, rubella virus, influenza virus, *Streptococcus* and *Staphylococcus* bacteria and others [9,10]. Infection-related occupational diseases are rarely reported in Poland in dentists [6,7,8]. The reason for this may be the lack of risk awareness, the neglect of the significance of the risk or the lack of reporting due to dentists’ unwillingness to report symptoms [11,12]. The basic methods of protecting workers from dental infectious diseases are vaccinations and the use of personal protective equipment [13,14].

The aim of the study was to analyze the knowledge of dentists about blood-borne infections and their attitudes towards patients infected with blood-borne viruses. It was also determined the types and frequency of the contact of dentists with potentially infectious material, their procedures for handling medical instrument waste, and their frequency of use of personal protective equipment.

## 2. Materials and Methods

The study was a part of a national survey on the exposure of healthcare workers in Poland to infectious material. According to the Central Statistical Office in Poland in 2014 there were 40,100 employed dentists, of which 97% were employed in the private sector, mostly in single offices, making it difficult to contact a large group of professionally active dentists.

Initially we conducted a pilot study designed to test the return rate of the questionnaire. We selected 50 dentist offices in Poland, to which the research tool was sent. Despite two reminders the study return rate was only 8% (only four completely filled questionnaires). Therefore, the authors decided to change the sampling method. We distributed 262 questionnaires to doctors attending a national conference for dentists organized by the Regional Chamber of Physicians. We thus obtained 188 completed questionnaires (a 73% response rate).

Surveys obtained by the random (from the pilot study) and the targeted (from the conference) approaches were used in the calculations. In total, we managed to collect 192 questionnaires from dentists working in various health care centers in Poland.

Dentists received an anonymous questionnaire consisting of 45 questions. The questions related to their frequency and type of contact with infectious materials during the entire career, their use of personal protective equipment, and their attitudes towards patients infected with blood-borne viruses, as well as their knowledge of such infections. In addition, the questions referred to injuries that happened during the last 12 months preceding the survey. In the questionnaire a short knowledge test has been also included focused in five statements, to which possible answers were: “true”, “false” or “I do not know”. The following questions asked were:

Q1. Hand disinfection can be replaced by the use of protective gloves.
Q2. In an emergency situation, the disinfection of hands is not required.
Q3. Approximately 60% of HBV infections among adults in Poland are nosocomial.
Q4. In the case of a single puncture by used needle, it is easier to become infected with HIV than HBV.
Q5. Tuberculosis infection is possible only by droplets.

The answers to the above questions constitute five independent variables, which measure the level of dentist’s knowledge. Descriptive statistics elements (frequency distributions) and analytical statistics were used. The analysis took into consideration four types of variables:

1. Dentist’s characteristics (five independent variables): gender, years of employment, place of employment, number of places of employment, personal situation.
2. Dentist’s attitudes (three dependent variables): anxiety, precaution, service refusal.
3. Type of exposure—contacts with potentially infectious material within 12 months preceding the survey (six dependent variables): contact through intact skin, contact through non-intact skin, contact through mucous membranes, contact through splashing on conjunctiva, needle puncture injury, deep needle puncture injury.
4. Dentist’s practice (one dependent variable): handling of used needles.

We considered the data set consisting of 10 dependent variables and 10 independent variables. We performed Pearson’s chi-squared and Cramér’s V tests of independence for each pair of dependent and independent variables to study the existence of stochastic relationship between variables describing the general population. Moreover, to evaluate the precision of estimated proportions we used the Jeffreys confidence intervals (see, for example, [15]). We run ordered logistic regressions to explain the risks of dentists’ contacts with potentially infectious material within 12 months preceding the survey. Statistical computations were performed using IBM SPSS Statistics 20 (IBM Corp., Armonk, NY, USA). The level of statistical significance was set at *p* ≤ 0.05.

The study protocol was approved by the Bioethics Committee of the Medical University of Lodz (Document No. RNN/163/14/KB of 11.02.2014) in full accordance with the World Medical Association Declaration of Helsinki. Written informed consent was obtained from all respondents before their participation in the survey.

## 3. Results

### 3.1. Characteristics of the Study Group

A large majority of the 192 dentists were women (*p* < 0.05), with a period of service longer than 5 years, employed in at least two places and mostly in large cities. Most dentists were satisfied with the location and the nature of the work (Table 1).

### 3.2. Frequency of Contact with Blood and Other Body Fluids

Despite a number of risk factors found at the dentists’ workplace, one-third of respondents did not remember being involved in a situation dangerous to his/her safety or health. Almost all dentists had frequent contact with potentially infectious material. In the current study, 83% of the dentists admitted that such contact occurred several times a day. Only two respondents reported no contact in their workplace with patients’ blood, body fluids, secretions, or excretions.

When asked about exposure to infectious material in the last 12 months preceding the survey, the most common answer was “several times” through intact skin and “never” for other situations (Table 2). It is worth noting that during one year, 27.6% of the dentists had contact with infectious material via damaged skin, and 28.1% of the dentists reported transmucosal contact. Half (54.7%) of the dentists reported splashing infectious material on the conjunctiva of the eye. Single superficial injury was reported by 60.4% of the dentists, and 16.7% of the dentists suffered deep laceration or needle puncture injuries. Risky contact with infectious material was more frequently reported by male dentists and by those dissatisfied with their job (variable: personal situation). The distribution of contact with infectious materials is presented in Table 2.

Although risky contact with potentially infectious material is considered relatively rare, only 20% of those surveyed (19% women vs. 27% men) reported zero needle punctures or injuries by other medical instruments during their professional careers. The risk of injury by medical sharp instruments and devices increased with more years of employment (*p* = 0.029).

The results of logistic regressions also confirmed that the years of employment is a significant variable (Table 3). The longer seniority, the less contact with intact skin, but significantly more cases of needle puncture injuries and more contacts with potentially infectious material through mucous membranes. Interestingly, the more the employee was pleased with the work the lower was the risk of contact with infectious material.

### 3.3. Applied Methods of Prevention

Most of the surveyed dentists (83%) felt that proper hygiene procedures and vaccination could protect workers from possible risks associated with their profession. Almost all (98%) were vaccinated against hepatitis B. It should be noted, however, that 78% of the dentists were vaccinated more than 5 years ago, and among them, 40% had never been tested for anti-HBs antibody levels.

Gloves were among the personal protective equipment most commonly used and were always used by 95% of the dentists. In addition, 37% of the dentists reported that sometimes (when appropriate) they wore two pairs of protective gloves. Visors, such as a mask, goggles and helmet, were never used by 6.5% of the dentists.

Although generally we have not found any major negligence in the use of personal protective equipment, as many as half of the dentists admitted that sometimes they discontinue use of protective equipment (e.g., take off protective gloves) to facilitate a dental service. Those behaviors were not related to any of the studied variables (*p* < 0.05).

The handling of used needles was also analyzed. Less than half of the dentists (45%) always placed the needle in a special container immediately after use, and 35% of the dentists admitted that they replaced protective cap on the needle prior to discarding. One-fifth reported that he/she usually acted properly, but occasionally replaced the protective cap on the needle, thus contributing to an increased risk of injury. Dentists with more years of employment more often handled used needles correctly (*p* = 0.016) (Table 4). In the group of dentists who reported replacing caps on contaminated needles, almost one-fifth (17%) of the dentists were injured during the replacing procedure.

All dentists reported that the offices in which they worked were equipped with special containers for infectious waste. Less than 5% reported that infectious waste containers were available but were not properly labeled. Additionally, 18% said they sometimes mistakenly toss infectious material into a wrong container. Among those who acknowledged doing so, the majority (60.5%) of the dentists made the mistake because they were rushed, and 14% of the dentists confused containers because of negligence. As many as 19% believed that the infectivity of the material was low.

Subjective self-assessment of compliance with hygiene procedures looked favorable; 77% of the dentists confirmed that the always followed procedures, and 21% of the dentists reported that they were occasional negligent, but the instances of neglect were rare.

### 3.4. Knowledge of Blood-Borne Infections and Their Prevention

One-fourth (27%) of the dentists answered all five questions correctly, which should be considered inadequate. They frequently (43%) answered four questions correctly. One-tenth of the dentists only correctly answered one or two questions. Differences in response rates did not depend on any of the examined variables.

Average value of correct answers given to all questions by respondents was 77%. Table 5 presents the structure of correct answers to particular questions. For example, that was 92% correct answers to the question “*In the case of a single puncture by used needle, it is easier to become infected with HIV than HBV*”, and only 57% correct answers to the question “*Tuberculosis infection is possible only by droplets*”. It is noteworthy that dentists with longer service and more experience gave fewer correct answers to questions. For example only 48% of dentists with longer seniority answered correctly that tuberculosis can infect not only by droplet. Younger dentists gave significantly more correct answers.

In the survey we asked about the source of knowledge. The knowledge (about the transmission and prevention of infectious diseases) of most dentists (80%) was based on information acquired during their university studies. Furthermore 73% of the dentists had improved their qualifications through various post-graduate refresher courses. Additionally, 53% of the dentists did so by reading current scientific journals. Two-thirds of the dentists reported that their employers do not organize training on post-exposure procedures. Most (86%) felt the need to increase their knowledge about the possibility of infection.

### 3.5. Attitudes of Dentists to Patients Infected with Blood-Borne Viruses

As many as 64% of the dentists were moderately apprehensive of being infected with HIV, HBV or HCV as a result of performing their job, and 17% of the dentists expressed a large concern in that respect (Table 6). Dentists who felt insecure at work and who were professionally unfulfilled were more often concerned about the likelihood of being infected while performing their professional duties (*p* = 0.002). Additionally, 22% of the dentists admitted that they knew in person a health care professional who had become infected as a result of occupational exposure.

Changing their behavior under the influence of concerns about their own health is a source of major anxiety among people dealing with patients on a daily basis. Almost all dentists (96%) confirmed that the need to help people infected with HIV, HBV or HCV caused them to be more cautious in dealing with those patients. Behavior changes were not affected by any of the analyzed variables. A large majority (70%) said that, to improve safety in the workplace, patients should be obliged to inform medical personnel about their HIV, HBV or HCV status. A similar proportion (71%) believed that a system of communication should be implemented in Poland to enable the exchange of information between health care facilities on whether a patient is infected with blood-borne viruses.

While 74% of the dentists have never refused to perform specific procedures in patients with known infections, 25% of the dentists refused to provide service to infected patients out of fear of jeopardizing their own health. Dentists from smaller towns more often refused to serve an infected patient (*p* = 0.004).

## 4. Discussion

In various studies [16,17,18,19,20], the authors raised the problem of the insufficient level of knowledge of dentists regarding the risk of transmission of blood-borne pathogens in dental offices and the legitimacy of the use of personal protective equipment. In the current study, we found that the level of knowledge of dentists about blood-borne infections and the risk of occupational infection was unsatisfactory, and its level varied. Dentists, in most cases, receive additional training through various courses, but unfortunately, those are rarely organized by the Polish public dental care system. This is likely because most dentists are self-employed (over 90% of Polish dentists work in the private sector), running single dental offices, hence they need to participate in commercially organized training courses and conferences. In Poland, the organizers of dental care at the national and provincial level do not have effective tools (positive—awards, and negative—penalties) to oblige and motivate dentists from the private sector to raise their qualifications. For this reason, dentists are based on the knowledge acquired during their studies and the level of knowledge decreases with age.

The vast majority of dentists apply protective clothing, such as aprons, gloves and face shields; however, nearly half reported that they sometimes remove protective clothing, for example gloves, to more easily perform procedures. The neglect in the proper sorting of infectious waste and the incorrect practice of replacing the protective cap onto the used needle was revealed. Replacing the needle cap is known for a long time to increase the risk of injury [21]. In our study, in the group of participants who acknowledged that they replaced the caps on the needle, one-fifth suffered needle puncture injuries.

The specific nature of the dentist’s job, such as the dentist’s inhalation zone shared with patient’s exhalation zone, as well as the use of high-speed tools producing large amounts of aerosols, saliva and blood splashing onto the dentist’s face, etc., creates multiple possibilities for contact with potentially infectious material [22,23]. In the current study 55% of the reported exposures involved splashes onto the ocular conjunctiva, similar to the figure reported by Shimojji et al. (60.3%) [23].

Hardie [24] demonstrated that most Canadian dentists feared the transfer of an infectious agent, and did not truly believe in the efficacy of the recommended preventive procedures [21]. The majority of Polish dentists (81%) are also afraid of being infected during work, and almost all (96%) admitted that they performed medical procedures more carefully with patients known to be infected with HIV, HBV or HCV. Only Arenas et al. [25] did not find changes in the behavior of dentists as a result of the awareness that the patient was infected. These findings may indicate a lack of the awareness that absolutely every patient should be treated as a potential source of infection. The patient himself is not obliged to inform that he/she is infected; therefore, in a job where there is a possibility of contact with potentially infectious material, the same safety precautions must be used for all patients. Whether there is a risk to the health or safety of others (doctors and patients) is evaluated by a physician taking care of the infected patient and is based on current medical evidence. As in the study by Gerbert [26], Polish dentists also understand the need to ensure dental care to infected patients, but would prefer not to be involved in that care themselves. One of the major alarming observation in the current study is that one-fourth dentists acknowledged that he/she had refused to provide service to an infected patient. This is a very important issue. On one hand, we believe that is probably a greater number of dentists who refuse treatment (negative behavior is difficult to assess through a survey—the limitation of this study). On the other hand, it is likely that the patient, who once denied treatment does not inform the next dentist that he is infected (expecting a similar negative reaction). A survey conducted in Denmark showed that most dentists were afraid of being infected, and in their opinion, HIV patients should be treated in specially adapted dental offices [27]. Similar to our results, most dentists believed that patients with HIV should be required to inform physicians of their status. Moreover, Polish dentists think that such information should be compulsorily transferred between different health institutions. Such practices would certainly jeopardize patients’ right to privacy and equal treatment. The mission of medical professionals is to help those with illness or injury and to save lives. In the treatment of dental patients, life-saving operations are very rare; therefore, we believe that a fair exchange of opinions on dentists’ concerns about and their attitudes towards infected patients is indispensable for the doctors to work safely and for the patients to feel comfortable.

## 5. Conclusions

Despite the widespread tolerance to people infected with blood-borne viruses and in spite of well-proven knowledge of the low risk of the infection of medical personnel from infected patients, Polish dentists continue to be prejudiced and concerned about their own health and more or less prone to refuse to treat infected patients. This attitude should imply the implementation of training in the field of pathogen transmission and the real risk of infection. A relatively high percentage (25%) of dentists refuse to treat a patient with an infection, posing a serious threat to public health.

## Figures and Tables

**Table 1:** Characteristics of the study group.

Characteristics of the Study Group	Number of Dentists	% Dentists (95% CI)
Gender		
Female	162	84.4 (78.9, 88.8)
Male	30	15.6 (10.9, 21.0)
Total	192	100.0
Years of employment		
<5	47	29.7 (23.3, 36.3)
5–15	49	25.5 (19.5, 31.9)
16–25	45	23.4 (17.6, 29.7)
>25	41	21.4 (15.8, 27.4)
Total	192	100.0
Place of employment		
village, small town	73	38.0 (31.3, 44.8)
big town	119	62.0 (55.0, 68.4)
Total	192	100.0
Number of places of employment		
1	81	42.2 (35.3, 49.0)
2 or more	111	57.8 (50.8, 64.4)
Total	192	100.0
Personal situation		
“I feel insecure; I don’t develop professionally”	34	17.7 (12.7, 23.3)
“I am professionally fulfilled, sure of development and employment in the future”	158	82.3 (76.6, 87.0)
Total	192	100.0

**Table 2:** Distribution of dentists’ contacts with potentially infectious material within 12 months preceding the survey.

Type of Exposure	Every Day		11 to 19 Times		2 to 10 Times		Once		Never	
	*n*	% (95% CI)	*n*	% (95% CI)	*n*	% (95% CI)	*N*	% (95% CI)	*n*	% (95% CI)
Contact through intact skin	19	9.9 (6.3, 14.7)	35	18.2 (13.3, 24.1)	67	34.9 (28.4, 41.8)	26	13.5 (9.3, 18.9)	45	23.4 (17.9, 29.8)
Contact through nonintact skin **^a^**	2	1.0 (0.2, 3.3)	6	3.1 (1.3, 6.3)	26	13.5 (9.3, 18.9)	19	9.9 (6.3, 14.7)	139	72.4 (65.8, 78.4)
Contact through mucous membranes **^a,b^**	11	5.7 (3.1, 9.7)	7	3.6 (1.6, 7.0)	30	15.6 (11.0, 21.3)	6	3.1 (1.3, 6.3)	138	71.9 (65.2, 77.9)
Contact through splashing on conjunctiva **^b^**	1	0.5 (0.1, 2.4)	15	7.8 (4.6, 12.2)	63	32.8 (26.5, 39.7)	26	13.5 (9.3, 18.9)	87	45.3 (38.4, 52.4)
Needle puncture injury	1	0.5 (0.1, 2.4)	2	1.0 (0.2, 3.3)	56	29.2 (23.1, 35.9)	57	29.7 (23.6, 36.4)	76	39.6 (32.9, 46.6)
Deep needle puncture injury **^a^**	0	0.0	2	1.0 (0.2, 3.3)	8	4.2 (2.0, 7.7)	22	11.5 (7.5, 16.5)	160	83.3 (77.6, 88.1)

**^a^** Statistically significant for the variable “personal situation in the first place of employment” at *p* < 0.05, Pearson’s chi-squared test and V-Cramer test; 95% CI—95% Confidence Interval; **^b^** Statistically significant for the variable “gender” at *p* < 0.05, Pearson’s chi-squared test and Cramér’s V tests.

**Table 3:** The risks (odds ratios (ORs)) of dentists’ contacts with potentially infectious material within 12 months preceding the survey.

Type of Exposure	Gender	Years of Employment	Place of Employment	Number of Places of Employment	Personal Situation
Contact through intact skin	ns	0.792 * (0.630, 0.995)	ns	ns	ns
Contact through nonintact skin	ns	ns	ns	0.564 **^+^** (0.291, 1.092)	0.237 ** (0.112, 0.503)
Contact through mucous membranes	ns	1.295 **^+^** (0.975, 1.721)	ns	ns	0.497 **^+^** (0.233, 1.061)
Contact through splashing on conjunctiva	ns	ns	ns	ns	ns
Needle puncture injury	ns	1.254 **^+^** (0.981, 1.603)	ns	ns	ns
Deep needle puncture injury	ns	ns	ns	ns	0.411 * (0.175, 0.965)

OR; 95% confidence intervals in brackets. **^+^** *p* < 0.1, * *p* < 0.05, ** *p* < 0.01, ns—not statistically significant.

**Table 4:** Handling of used needles by dentists.

Characteristic of Dentists			Handling of Used Needles			Total
			Always, Immediately after Use I Place the Needle in Special Container	Usually I Place the Needle in Special Container, But Sometimes I Replace the Protective Cap	I Always Replace the Protective Cap onto the Needle	
Years of employment	≤5	*n*	17	13	27	57
		%	29.8	22.8	47.4	100.0
		95% CI	(19.2, 42.5)	(13.4, 34.9)	(34.8, 60.2)	
	>5	*n*	70	25	40	135
		%	51.9	18.5	29.6	100.0
		95% CI	(43.5, 60.2)	(12.7, 25.7)	(22.4, 37.7)	
Total		*n*	87	38	67	192
		%	45.3	19.8	34.9	100.0
		95% CI	(38.4, 52.4)	(14.6, 25.9)	(28.4, 41.8)	

Statistically significant at *p* < 0.05, Pearson’s chi-squared test and Cramér’s V tests.

**Table 5:** Number and percentage (%) of correct answers in the test of knowledge, depending on the variables examined.

**Variables Question**	**Total**		**Gender**				**Years of Employment**				**Place of Employment**			
			**Female**		**Male**		**<5**		**≥5**		**Village, Small Town**		**Big Town**	
	***N* = 192**	**100%**	***N* = 162**	**100%**	***N* = 30**	**100%**	***N* = 57**	**100%**	***N* = 135**	**100%**	***N* = 73**	**100%**	***N* = 119**	**100%**
Question 1 **^a^**	186	96.9	157	96.9	29	96.7	57	100	129	95.6	72	98.6	114	95.8
Question 2 **^b^**	141	73.4	120	74.1	21	70.0	43	75.4	98	72.6	49	67.1	92	77.3
Question 3 **^c^**	131	68.2	107	66.0	24	80.0	42	73.7	89	65.9	47	64.4	84	70.6
Question 4 **^d^**	176	91.7	150	92.6	26	86.7	53	93.0	123	91.1	67	91.8	109	91.6
Question 5 **^e^**	109	56.8	91	56.2	18	60.0	44 *	77.2	65 *	48.1	37	50.7	72	60.5
**Variables Question**	**Total**		**Number of Places of Employment**				**Personal Situation**							
			**1**		**2 or More**		**“I Feel Insecure; I Don’t Develop Professionally”**		**“I Am Professionally Fulfilled, Sure of Development and Employment in the Future”**					
	***N* = 192**	**100%**	***N* = 81**	**100%**	***N* = 111**	**100%**	***N* = 34**	**100%**	***N* = 158**	**100%**				
Question 1 **^a^**	186	96.9	81 *	100	105 *	94.6	34	100	15	96.2				
Question 2 **^b^**	141	73.4	60	74.1	81	73.0	28	82.4	113	71.5				
Question 3 **^c^**	131	68.2	49 *	60.5	82 *	73.9	24	70.6	107	67.7				
Question 4 **^d^**	176	91.7	75	92.6	101	91.0	29	85.3	147	93.0				
Question 5 **^e^**	109	56.8	48	59.3	61	55.0	23	67.6	86	54.4				

**^a^** Hand disinfection can be replaced by the use of protective gloves; **^b^** In an emergency situation, the disinfection of hands is not required; **^c^** Approximately 60% of HBV infections among adults in Poland are nosocomial; **^d^** In the case of a single puncture by a used needle, it is easier to become infected with HIV than HBV; **^e^** Tuberculosis infection is possible only by droplets. * Statistically significant at *p* < 0.05, chi-squared test.

**Table 6:** Attitudes of dentists towards patients infected with blood-borne viruses.

Attitudes towards Infected Patients		Gender				Years of Employment				Place of Employment				Personal Situation			
		F		M		≤5		>5		Village, Small Town		Big Town		“I Feel Insecure; I Don’t Develop Professionally”		“I Am Professionally Fulfilled, Sure of Development and Employment in the Future”	
		*n*	% (95% CI)	*N*	% (95% CI)	*n*	% (95% CI)	*n*	% (95% CI)	*n*	% (95% CI)	*n*	% (95% CI)	*n*	% (95% CI)	*n*	% (95% CI)
Fear of infection as a result of occupational exposure.	No	31	19.1 (13.5, 25.4)	6	20.0 (8.0, 34.7)	9	15.8 (7.6, 25.8)	28	20.7 (14.4, 27.7)	17	23.3 (14.4, 33.1)	20	16.8 (10.7, 23.8)	2 *	5.9 * (0.7, 15.3)	35 *	22.2 * (16.0, 28.8)
	Yes, moderately	101	62.3 (54.8, 69.2)	22	73.3 (56.5, 85.3)	40	70.2 (57.8, 80.1)	83	61.5 (53.2, 69.0)	44	60.3 (48.9, 70.3)	79	66.4 (57.7, 74.0)	20 *	58.8 * (42.1, 72.8)	103 *	65.2 * (57.6, 72.0)
	Yes, very much	30	18.5 (12.9, 24.7)	2	6.7 (0.8, 17.2)	8	14.0 (6.4, 23.7)	24	17.8 (11.8, 24.5)	12	16.4 (8.9, 25.4)	20	16.8 (10.7, 23.8)	12 *	35.3 * (20.4, 50.5)	20 *	12.7 * (8.0, 18.1)
Is the dentist to be more careful when dealing with infected patients?	No	6	3.7 (1.4, 7.1)	2	6.7 (0.8, 17.2)	1	1.8 (0.0, 6.3)	7	5.2 (2.1, 9.4)	3	4.1 (0.9, 9.5)	5	4.2 (1.4, 8.4)	0	0.0 -	8	5.1 (2.2, 8.9)
	Yes, a little more	40	24.7 (18.4, 31.4)	8	26.7 (12.7, 42.3)	16	28.1 (17.3, 39.7)	32	23.7 (16.9, 31.0)	18	24.7 (15.5, 34.6)	30	25.2 (17.9, 33.1)	7	20.6 (9.0, 34.5)	41	25.9 (19.4, 32.8)
	Yes, definitely more	116	71.6 (64.4, 77.8)	20	66.7 (49.2, 80.1)	40	70.2 (57.8, 80.1)	96	71.1 (63.2, 77.9)	52	71.2 (60.4, 80.1)	84	70.6 (62.1, 77.8)	27	79.4 (64.5, 89.3)	109	69.0 (61.6, 75.5)
Is there fear of infection, causing the dentist not to treat the patient?	No, never	117	72.2 (65.1, 78.4)	25	83.3 (68.3, 92.3)	47 *	82.5 * (71.7, 90.0)	95 *	70.4 * (62.4, 77.2)	46 *	63.0 * (51.7, 72.8)	96 *	80.7 * (73.1, 86.6)	22	64.7 (48.2, 77.8)	120	75.9 (69.0, 81.8)
	Yes, but only a few times	42	25.9 (19.5, 32.7)	5	16.7 (5.8, 30.7)	10 *	17.5 * (8.9, 27.9)	37 *	27.4 * (20.2, 35.0)	27 *	37.0 * (26.4, 47.7)	20 *	16.8 * (10.7, 23.8)	11	32.4 (18.0, 47.5)	36	22.8 (16.6, 29.4)
	Yes, many times	3	1.9 (0.4, 4.4)	0	0.0	0 *	0.0 *	3 *	2.2 * (0.5, 5.2)	0 *	0.0 *	3 *	2.5 * (0.5, 5.9)	1	2.9 (0.1, 10.3)	2	1.3 (0.2, 3.5)

* Statistically significant at *p* < 0.05, chi-squared test and Cramér’s V test; 95% CI—95% Confidence Interval.

## References

[1] Leggat P.A., Kedjarune U., Smith D.R. (2007). Occupational health problems in modern dentistry: A review. Ind. Health.

[2] Gonzalez Y.M. (1998). Occupational diseases in dentistry. Introduction and epidemiology. N. Y. State Dent. J..

[3] Al-Sayagh G.D. (2006). The occupational hazards and diseases among dentists in Mosul City: Musculoskeletal pain, eye problem and hepatitis. Al-Rafidain Dent. J..

[4] Leggat P.A., Smith D.R. (2006). Musculoskeletal disorders self-reported by dentists in Queensland, Australia. Aust. Dent. J..

[5] Alexopoulus E.C., Stathi I.C., Charizani F. (2004). Prevalence of musculoskeletal disorders in dentists. BMC Musculoskel. Disord..

[6] Szeszenia-Dąbrowska N., Wilczyńska U., Sobala W. (2013). Occupational Diseases in Poland in 2012.

[7] Szeszenia-Dąbrowska N., Wilczyńska U., Sobala W. (2014). Occupational Diseases in Poland in 2013.

[8] Szeszenia-Dąbrowska N., Wilczyńska U., Sobala W. (2015). Occupational Diseases in Poland in 2014.

[9] Araujo M.W., Andreana S. (2002). Risk and prevention of transmission of infectious diseases in dentistry. Quintessence Int..

[10] Singh A., Purohit B.M., Bhambal A., Saxena S., Singh A., Gupta A. (2011). Knowledge, attitudes, and practice regarding infection control measures among dental students in central India. J. Dent. Educ..

[11] Sohn S., Eagan J., Sepkowitz K. (2004). Safety-engineered device implementation: Does it introduce bias in percutaneous injury reporting?. Infect. Control Hosp. Epidemiol..

[12] Ayatollahi J. (2006). Needle-stick injuries in a general hospital: Continuing risk and under reporting. Ann. Iran. Med..

[13] Centers for Diseases Control and Prevention (2016). Summary of Infection Prevention Practices in Dental Settings. Basic Expectations for Safe Care.

[14] Centers for Diseases Control and Prevention (2003). Guidelines for Infection Control in Dental Health-Care Settings—2003.

[15] Brown L.D., Cai T.T., DasGupta A. (2001). Interval estimation for a binomial proportion. Stat. Sci..

[16] Askarian M., Mirzaei K., Barry Cookson B. (2007). Knowledge, attitudes, and practice of Iranian dentists with regard to HIV-related disease. Infect. Control Hosp. Epidemiol..

[17] Kabir A., Tabatabaei S.V., Khaleghi S., Agah S., Faghihi Kashani A.H., Moghimi M., Habibi Kerahroodi F., Alavian S.E., Alavian S.M. (2010). Knowledge, attitudes and practice of Iranian medical specialists regarding hepatitis B and C. Hepat. Mon..

[18] Chen M.Y., Fox E.F., Rogers C.A. (2001). Post-exposure prophylaxis for human immunodeficiency virus: Knowledge and experience of junior doctors. Sex. Transm. Infect..

[19] Shaghaghian S., Pardis S., Mansoori Z. (2014). Knowledge, attitude and practice of dentists towards prophylaxis after exposure to blood and body fluids. Int. J. Occup. Environ. Med..

[20] Ebrahimi M., Ajami B.M., Rezaeian A.R. (2012). Longer years of practice and higher education levels promote infection control in Iranian dental practitioners. Iran. Red Crescent Med. J..

[21] Jagger J., Hunt E., Brand-Elnaggar J., Pearson R.D. (1988). Rates of needle-stick injury caused by various devices in a university hospital. N. Engl. J. Med..

[22] Harrel S.K., Molinari J. (2004). Aerosols and splatter in dentistry: A brief review of the literature and infection control implications. J. Am. Dent. Assoc..

[23] Shimoji S., Ishihama K., Yaamada H., Okayama M., Yasuda K., Shibutani T., Ogasawara T., Miyazawa H., Furusawa K. (2010). Occupational safety among dental health-care workers. Adv. Med. Educ. Pract..

[24] Hardie J. (1992). The attitudes and concerns of Canadian dental health care workers toward infection control and the treatment of AIDS patients. J. Can. Dent. Assoc..

[25] Arenas M., Sánchez-Payá J., Barril G., García-Valdecasas J., Gorriz J.L., Soriano A., Antolin A., Lacueva J., García S., Sirvent A. (2005). A multicentric survey of the practice of hand hygiene in haemodialysis units: Factors affecting compliance. Nephrol. Dial. Transplant..

[26] Gerbert B. (1987). AIDS and infection control in dental practice: Dentist’ attitudes, knowledge, and behavior. J. Am. Dent. Assoc..

[27] Scheutz F. (1998). Dental care of HIV-infected patients: Attitudes and behavior among Danish dentists. Commun. Dent. Oral Epidemiol..

